# Supplementary material for: Persistence of the hepatic benefits of high-intensity interval training (HIIT) during detraining despite body weight regain in mice
Source: PLoS One. 2026 Feb 17;21(2):e0342671. doi: 10.1371/journal.pone.0342671 (PMC12912586; doi:10.1371/journal.pone.0342671)
Supplement: S1 File — (DOCX) [file pone.0342671.s001.docx]

**Supporting information**

**S1** - **The images included in this file correspond to the original Western blot membranes used for protein expression analysis.**

All experiments were performed in biological triplicates, using three independent membranes for each analyzed protein to achieve the final sample size (*n*).

File names follow the pattern *protein–membrane–replicate*, where M1, M2, and M3 correspond to independent membranes, and a, b, and c indicate individual biological replicates within each membrane (e.g., β-ACTIN *M1a, M1b, M1c; M2a, M2b, M2c; M3a, M3b, M3c*). All images are presented as originally acquired.

- β-ACTIN *M1a*


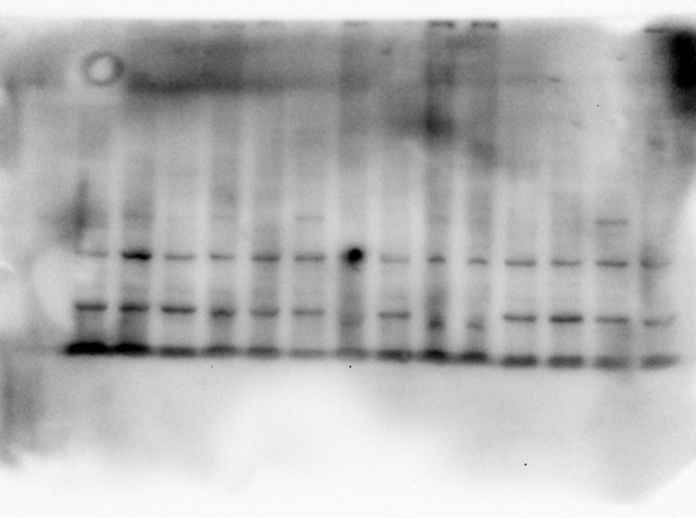


- β-ACTIN *M1b*


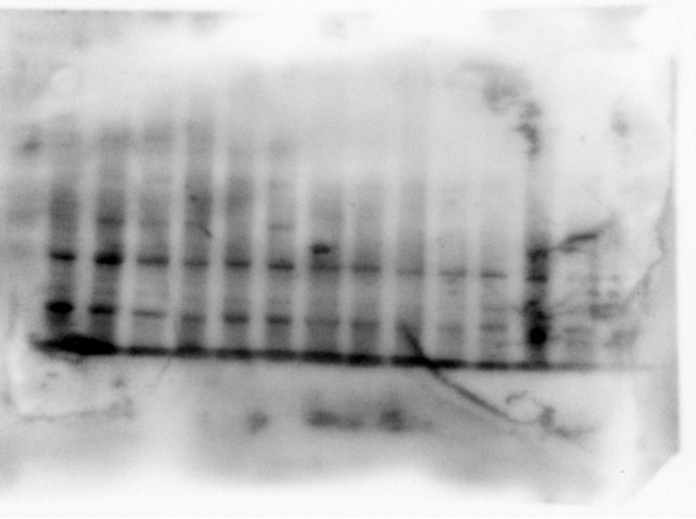


*-* β-ACTIN *M1c*


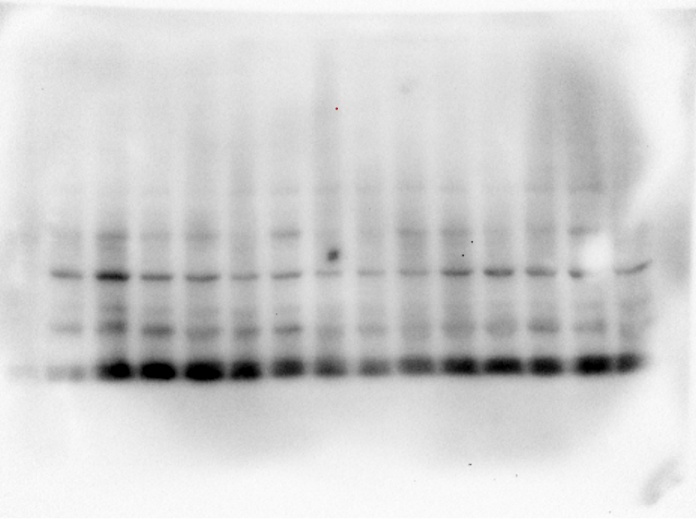


- β-ACTIN *M2a*


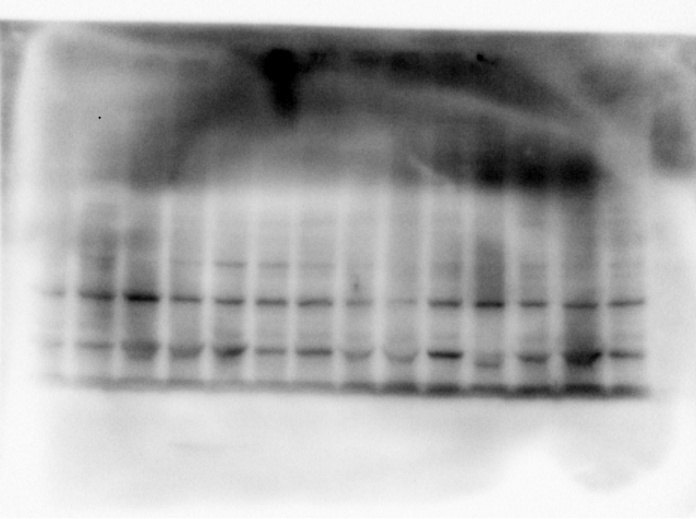


- β-ACTIN *M2b*


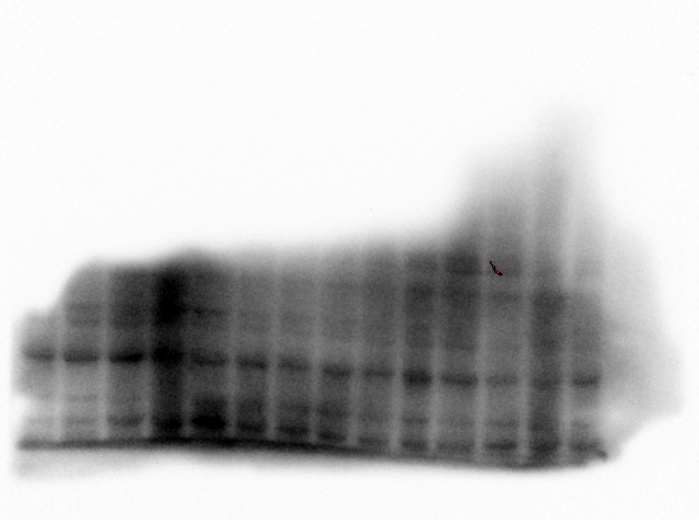


*-* β-ACTIN *M2c*


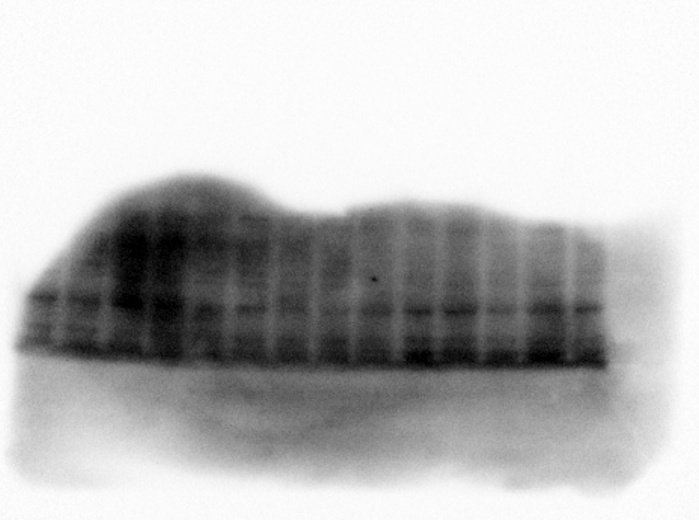


- β-ACTIN *M3a*

*
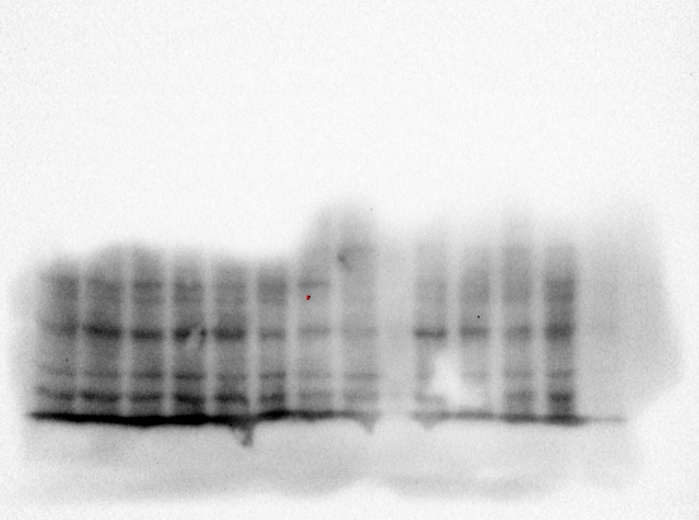
*

- β-ACTIN *M3b*

*
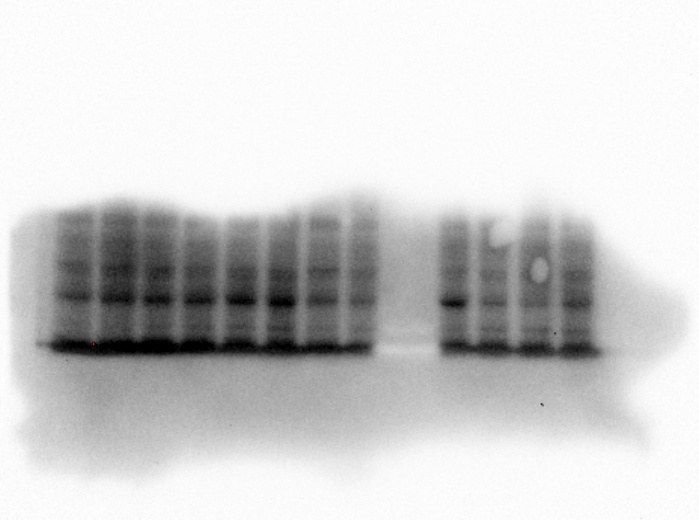
*

- β-ACTIN *M3c*

*
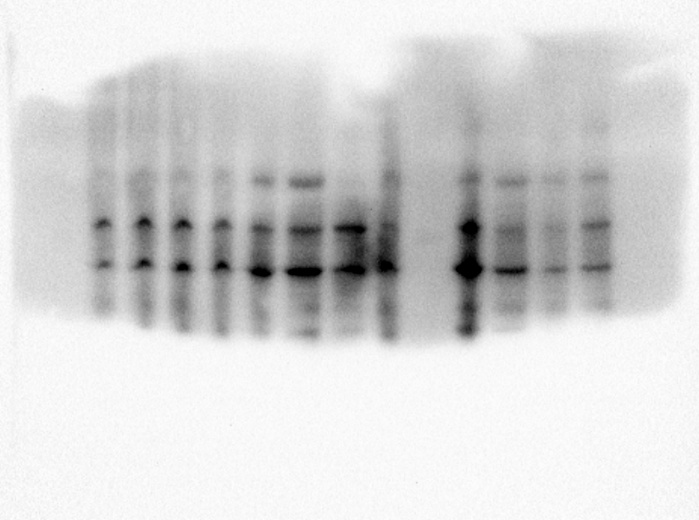
*

- ACOX *M1a*


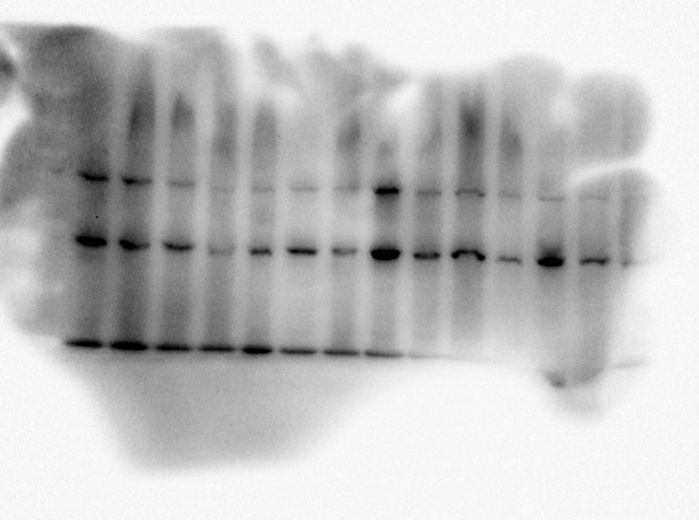


- ACOX *M1b*


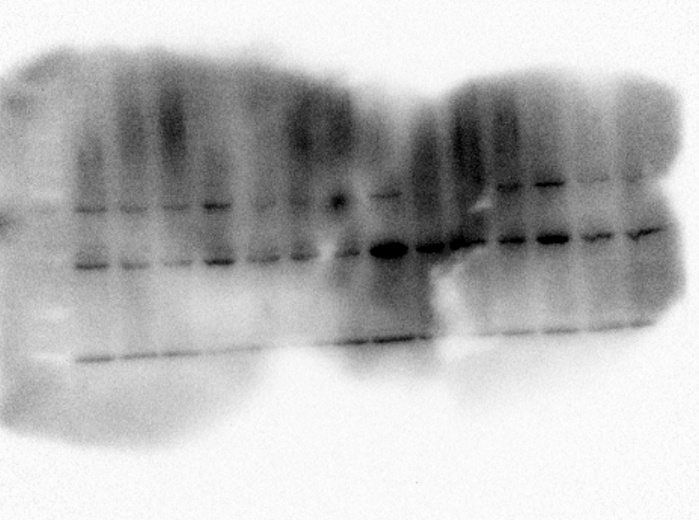


*- ACOX M1c*


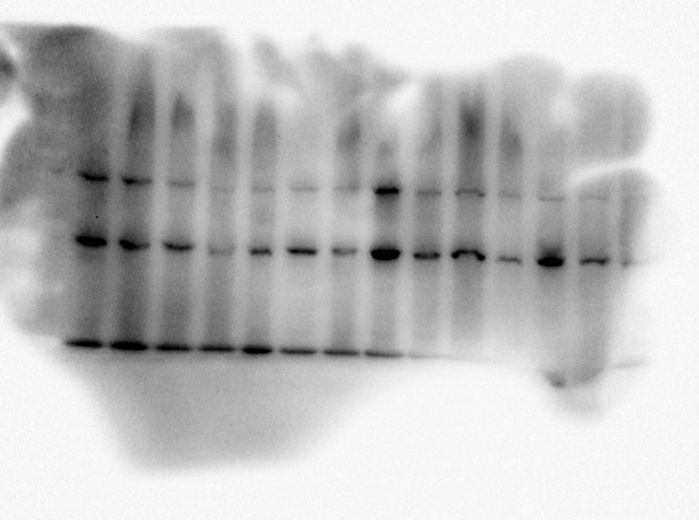


- ACOX *M2a*


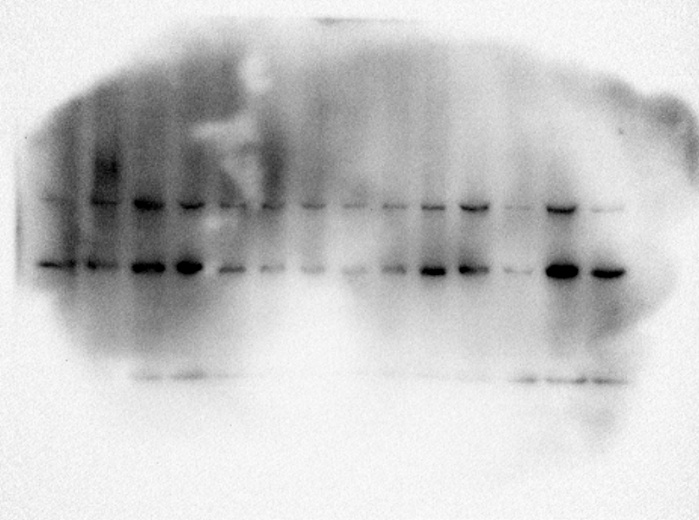


- ACOX *M2b*


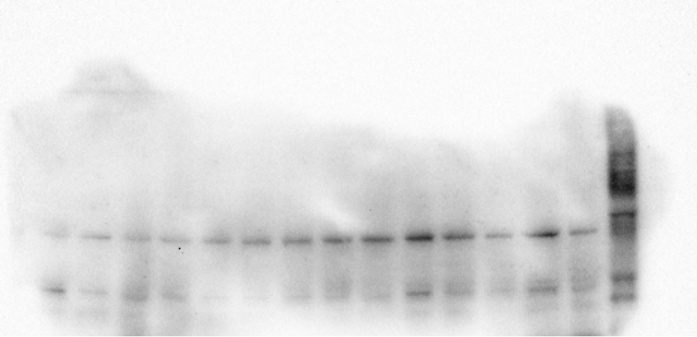


- ACOX *M2c*


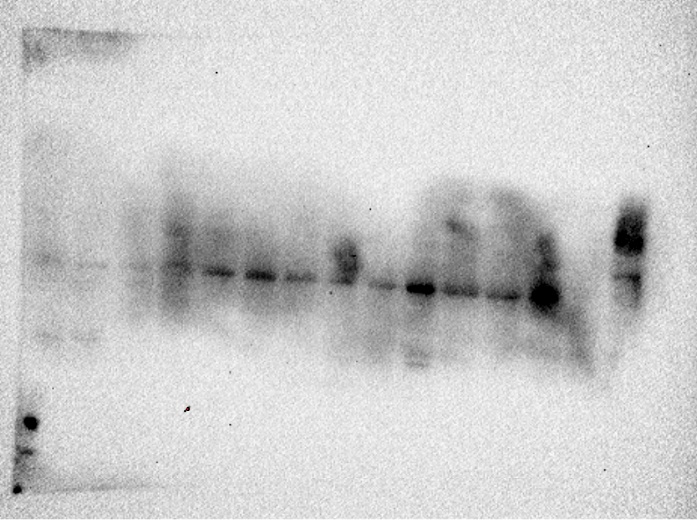


- ACOX *M3a*

*
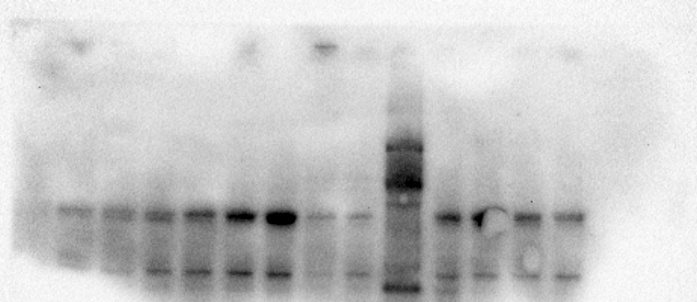
*

- ACOX *M3b*


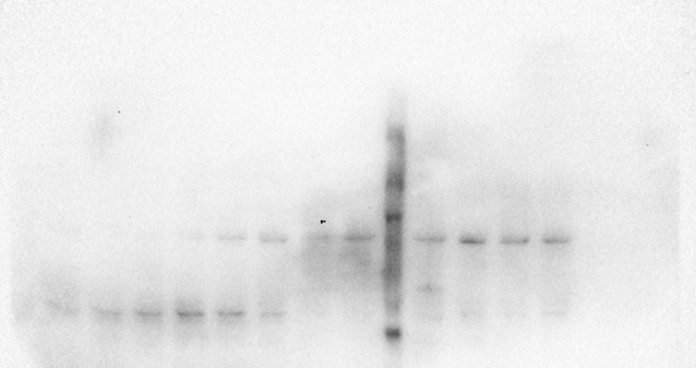


- ACOX *M3c*


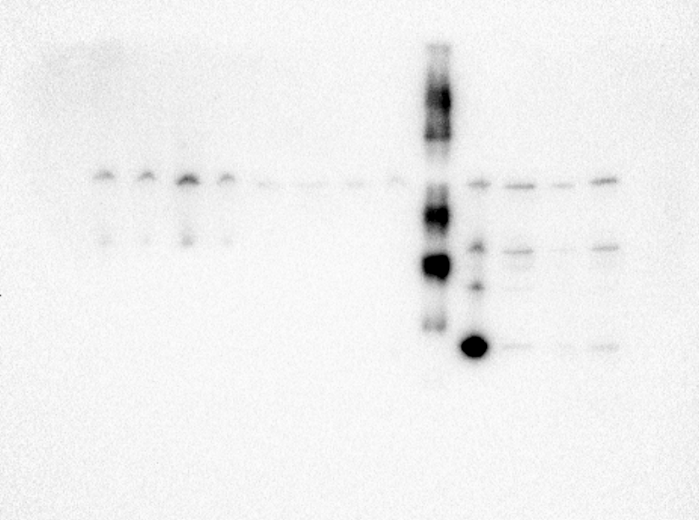


- CHREBP *M1a*


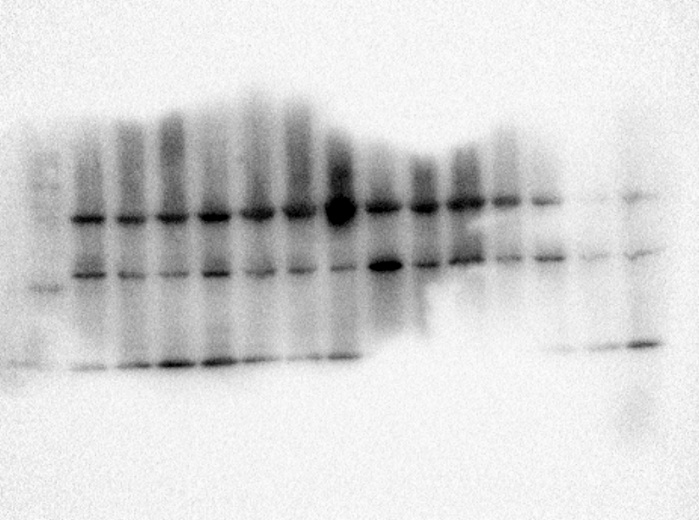


- CHREBP *M2a*


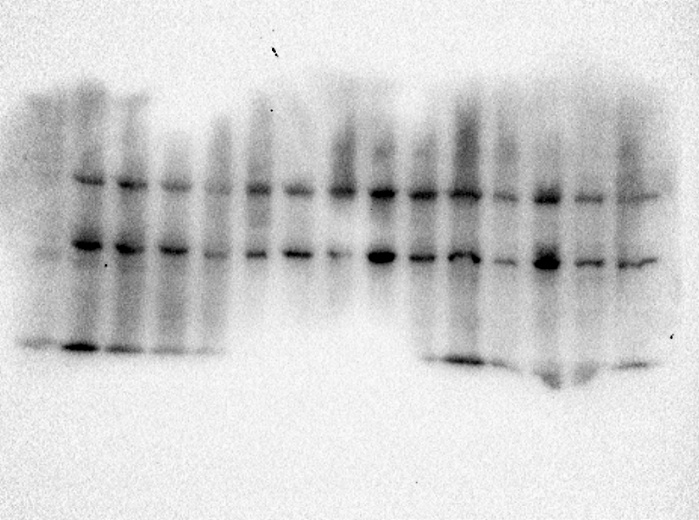


- CHREBP *M3a*

*
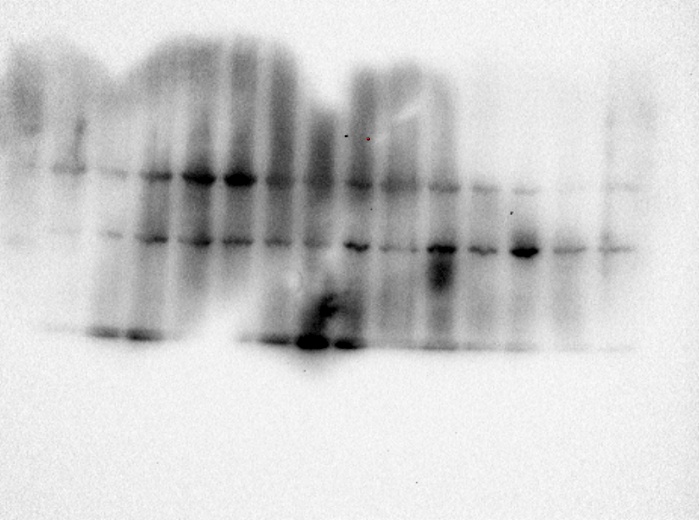
*

- CHREBP *M2a*


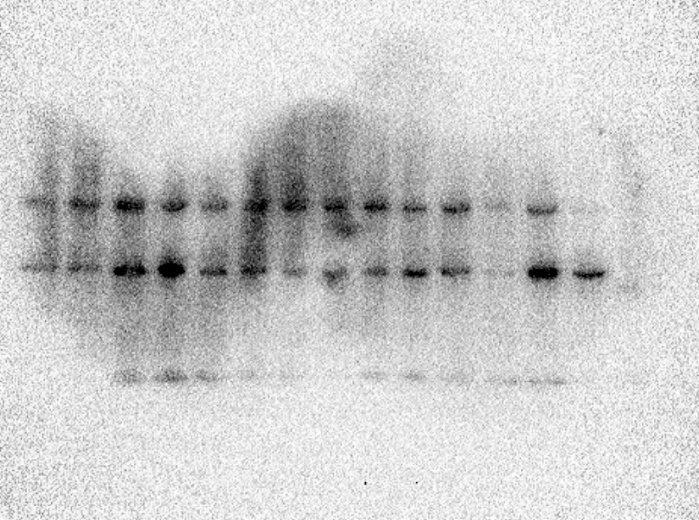


- CHREBP *M2b*

*
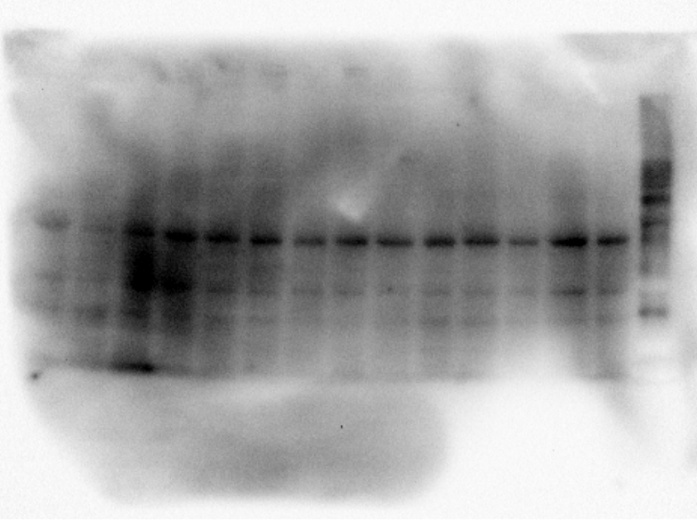
*

- CHREBP *M2c*

*
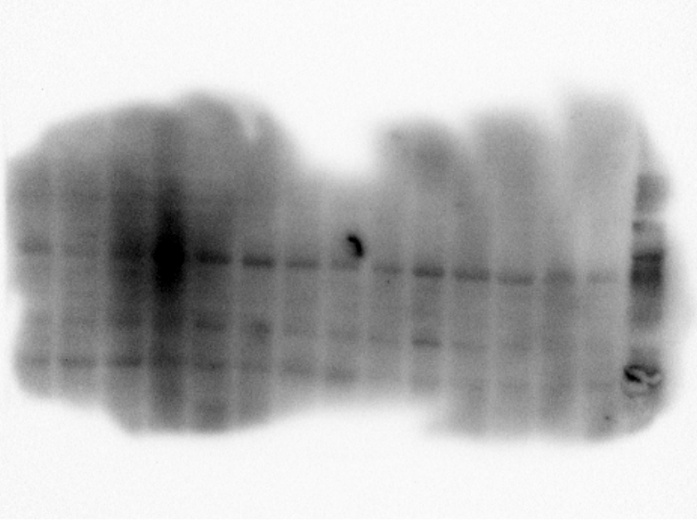
*

- CHREBP *M3a*

*
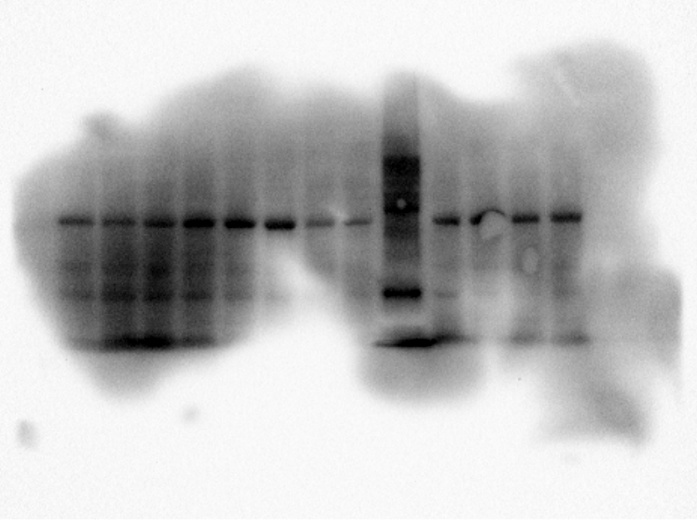
*

- CHREBP *M3b*

*
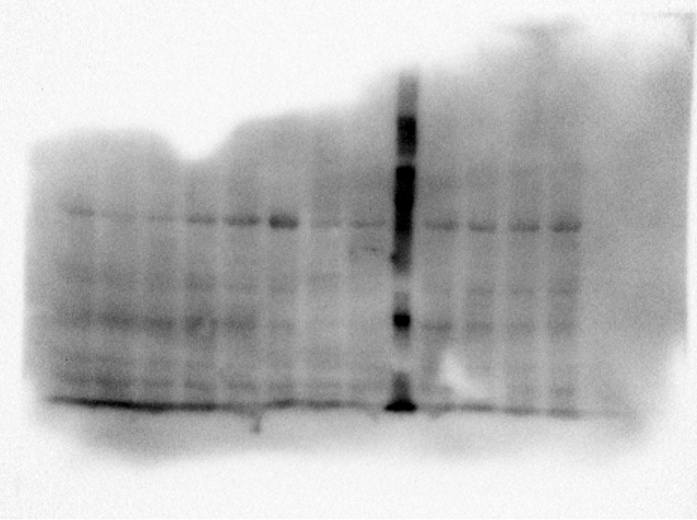
*

- CHREBP *M3c*

*
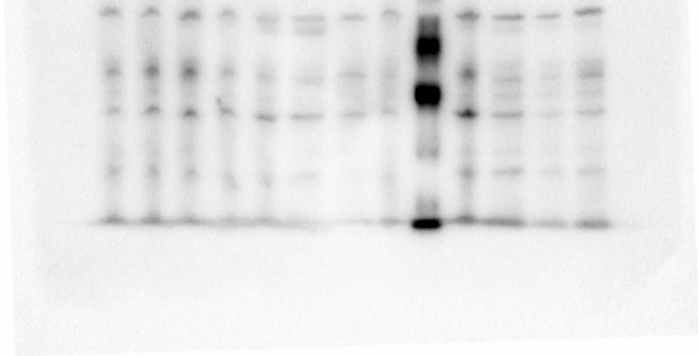
*

- GRP *M1a*

*
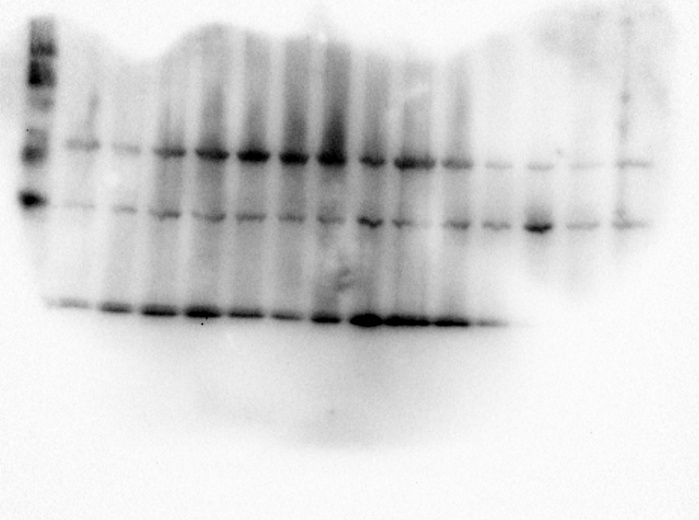
*

- GRP *M1b*

*
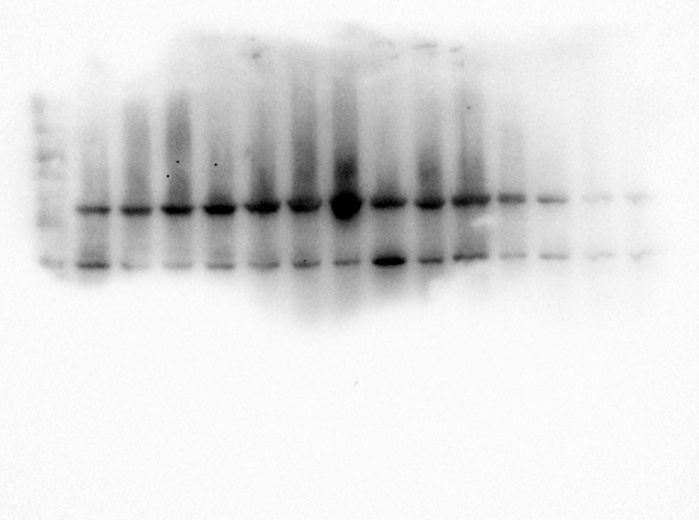
*

- GRP *M1c*

*
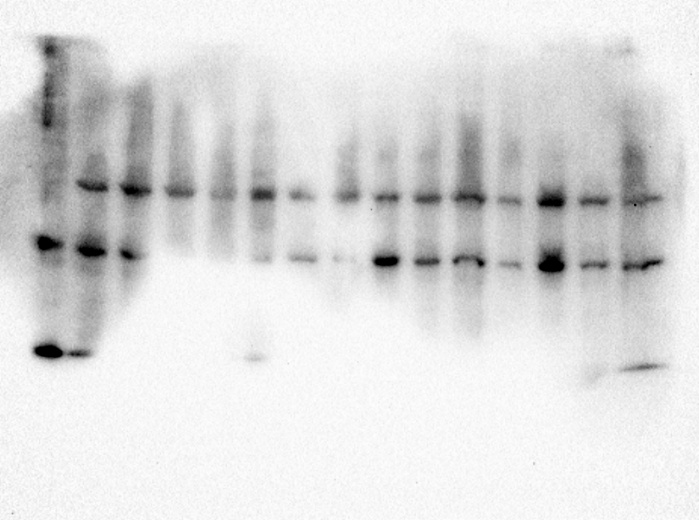
*

- GRP *M2a*

*
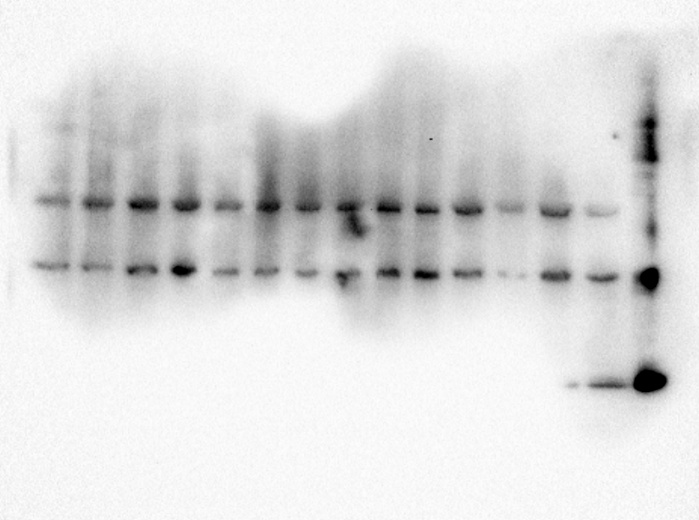
*

- GRP *M2b*


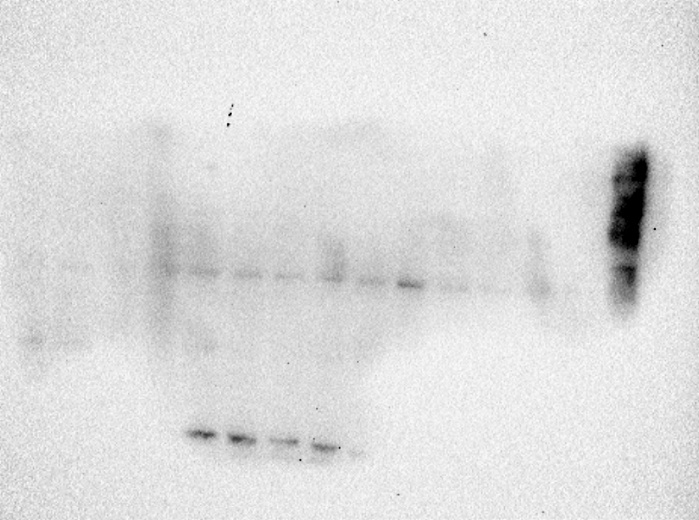


- GRP *M2c*


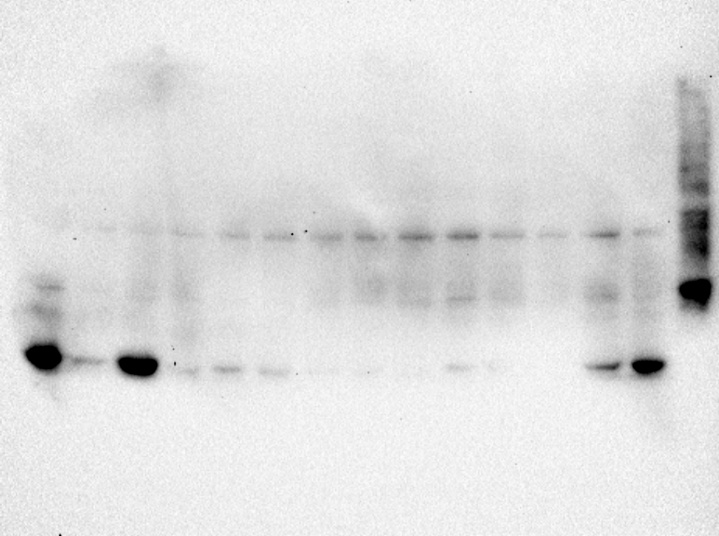


- GRP *M3a*


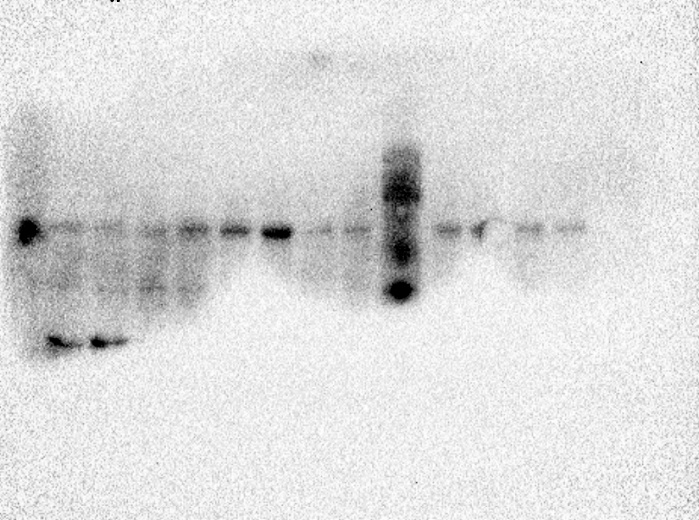


- GRP *M3b*

*
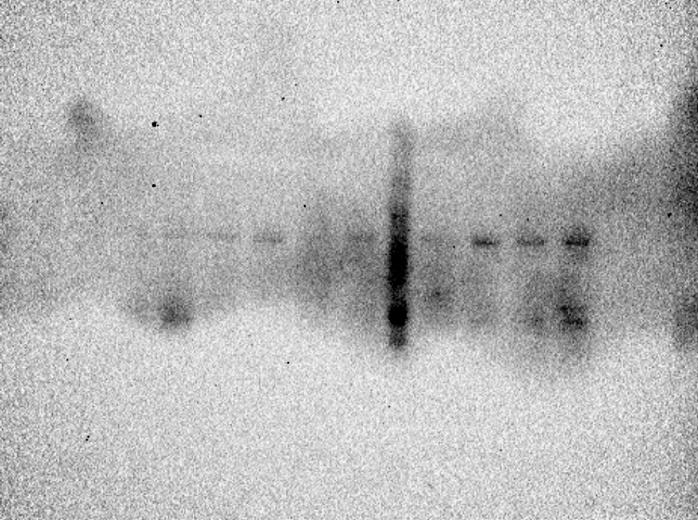
*

- GRP *M3c*

*
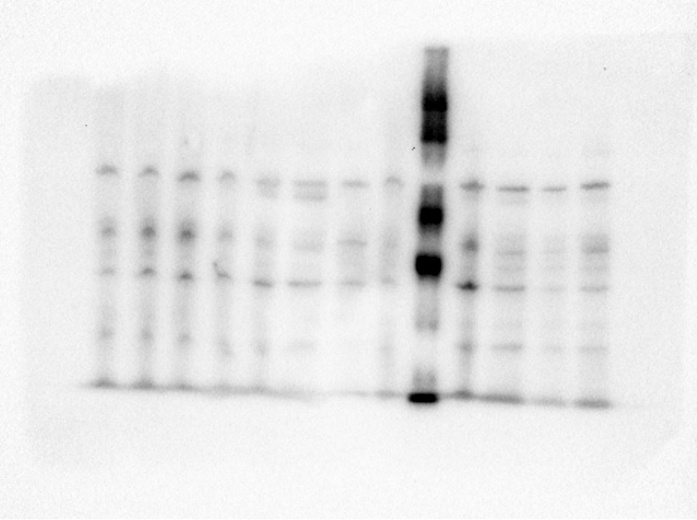
*

- SREBP *M1a*

*
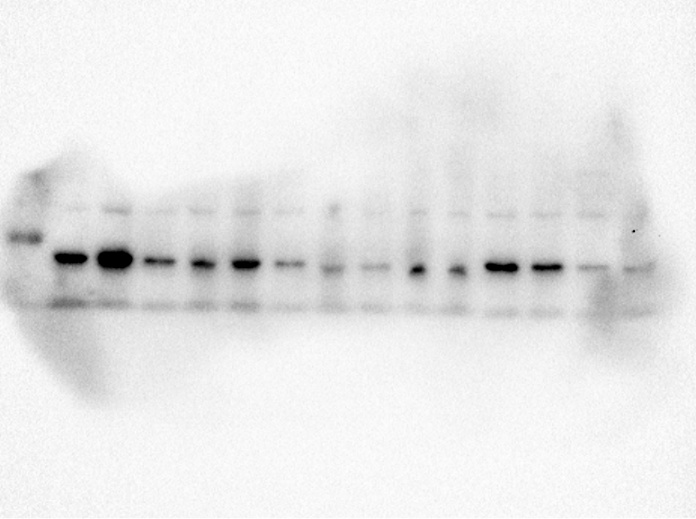
*

- SREBP *M1b*

*
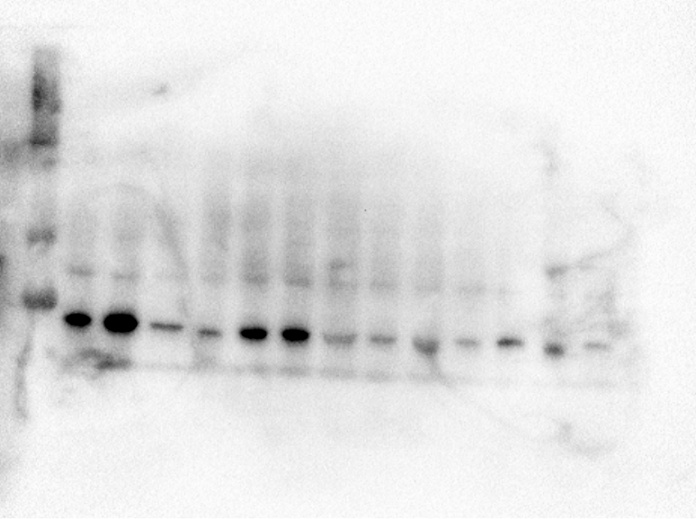
*

- SREBP *M1c*

*
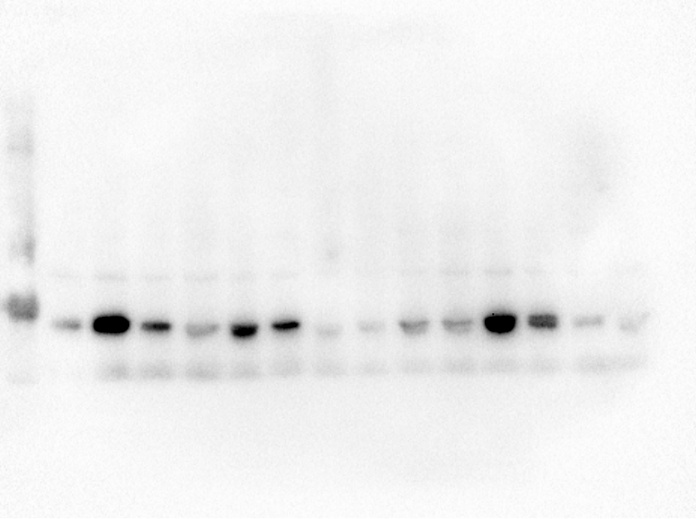
*

- SREBP *M2a*


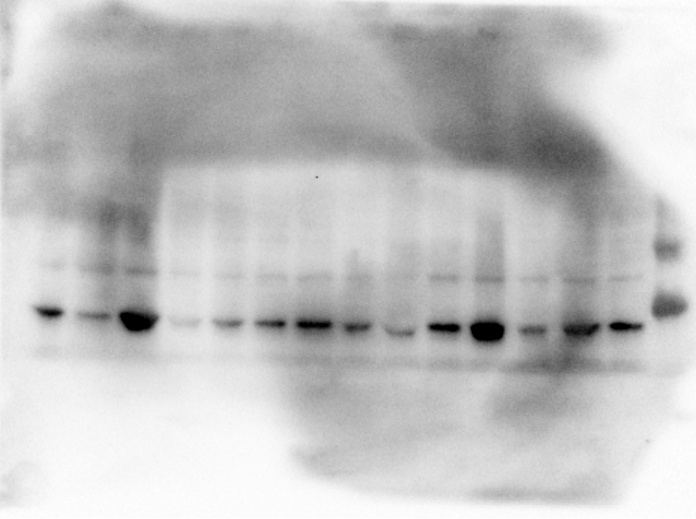


- SREBP *M2b*

*
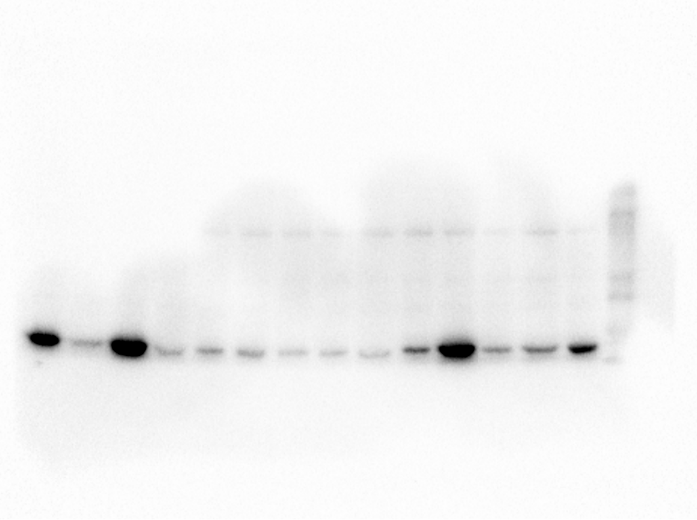
*

- SREBP *M2c*

*
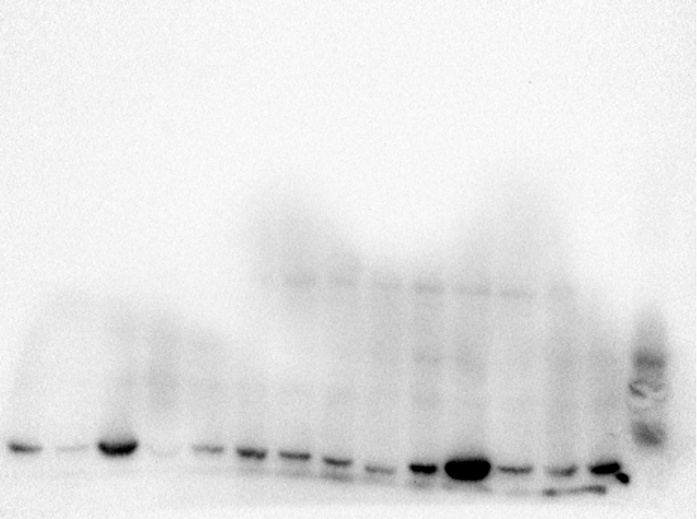
*

- SREBP *M3a*

*
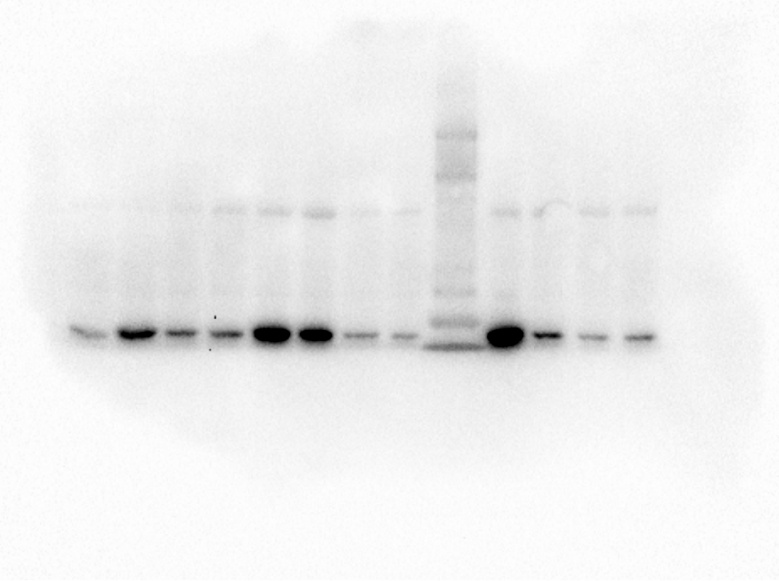
*

- SREBP *M3b*

*
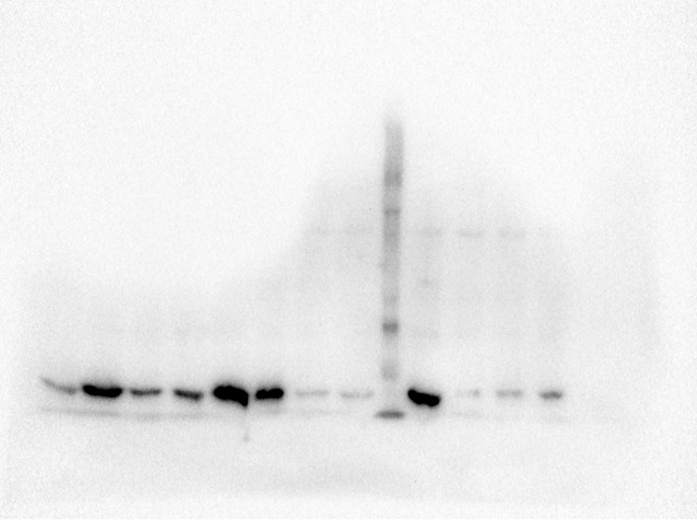
*

- SREBP *M3c*

*
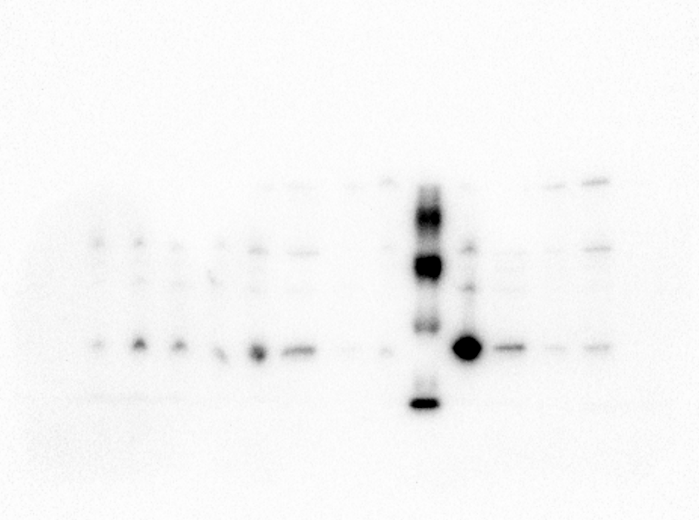
*
